# Healthcare Utilization, Costs, and Cost-Effectiveness of Patients Undergoing Laparoscopic and Open Hemihepatectomy: A Secondary Analysis of the ORANGE II PLUS Randomized Controlled, Phase 3, Superiority Trial

**DOI:** 10.1245/s10434-025-18779-4

**Published:** 2025-12-12

**Authors:** Bram Olij, Gabriela Pilz da Cunha, Merel Kimman, Francesca Ratti, Mohammad Abu Hilal, Roberto I. Troisi, Robert P. Sutcliffe, Marc G. Besselink, Somaiah Aroori, Krishna V. Menon, Bjørn Edwin, Mathieu D’Hondt, Valerio Lucidi, Tom F. Ulmer, Rafael Díaz-Nieto, Zahir Soonawalla, Steve White, Gregory Sergeant, Mariëlle M. E. Coolsen, Christoph Kuemmerli, Vincenzo Scuderi, Frederik Berrevoet, Aude Vanlander, Ravi Marudanayagam, Pieter J. Tanis, Maxime J. L. Dewulf, Robert S. Fichtinger, Zina B. Eminton, Ulf P. Neumann, Lloyd Brandts, Siân A. Pugh, Åsmund A. Fretland, John N. Primrose, Ronald M. van Dam

**Affiliations:** 1 Department of Surgery, Maastricht University Medical Centre+, Maastricht, The Netherlands; 2 GROW—School for Oncology and Developmental Biology, Maastricht University, Maastricht, The Netherlands; 3 Department of Surgery, Amsterdam UMC, Location University of Amsterdam, Amsterdam, The Netherlands; 4 Department of Clinical Epidemiology and Medical Technology Assessment, Maastricht University Medical Centre+, Maastricht, The Netherlands; 5 Hepatobiliary Surgery Division, IRCCS San Raffaele Hospital, Milan, Italy; 6 Department of Surgery, School of Medicine, the University of Jordan, Amman, Jordan; 7 University Surgery and Perioperative and Critical Care Theme, NIHR Southampton Biomedical Research Centre, University Hospital Southampton/University of Southampton, Southampton, UK; 8 Division of HPB, Minimally Invasive and Robotic Surgery, Department of Clinical Medicine and Surgery, Transplantation Service, Federico II University, Naples, Italy; 9 HPB and Liver Transplant Unit, University Hospitals Birmingham NHS Trust, Birmingham, UK; 10 Cancer Centre Amsterdam, Amsterdam, The Netherlands; 11 Department of Surgery, Plymouth Hospitals NHS Trust, Plymouth, UK; 12 Department of Liver Transplant and HPB Surgery, Institute of Liver Studies, King’s College Hospital NHS Foundation Trust, London, UK; 13 Intervention Centre and Department of Hepatic, Pancreatic and Biliary Surgery, Oslo University Hospital and Institute of Clinical Medicine, University Hospital of Oslo, Oslo, Norway; 14 Department of Digestive and Hepatobiliary/Pancreatic Surgery, AZ Groeninge, Kortrijk, Belgium; 15 Department of Digestive Surgery, Unit of Hepatobiliary Surgery and Transplantation, Hôpitaux Universitaires de Bruxelles, Hôpital Erasme, Brussels, Belgium; 16 Department of Surgery and Transplantation, University Hospital RWTH Aachen, Aachen, Germany; 17 Department of Surgery, University Hospital Essen, Essen, Germany; 18 Department of Hepato-Biliary Surgery, Aintree University Hospital NHS Foundation Trust, Liverpool, UK; 19 Department of Surgery, Oxford University Hospitals NHS Foundation Trust, Oxford, UK; 20 Department of Surgery, Newcastle Upon Tyne Hospitals NHS Foundation Trust, Newcastle upon Tyne, UK; 21 Department of Digestive and Hepatobiliary/Pancreatic Surgery, Jessa Hospital, Hasselt, Belgium; 22 Faculty of Medicine and Health Sciences, UHasselt, Hasselt, Belgium; 23 Department of General, HPB and Liver Transplantation Surgery, Ghent University Hospital, Ghent, Belgium; 24 Department of Surgery, Free University Hospital, AZ Jette Hospital, Brussels, Belgium; 25 Southampton Clinical Trials Unit, University of Southampton, Southampton, UK; 26 Department of Oncology, Addenbrooke’s Hospital, Cambridge, UK

**Keywords:** Cost-effectiveness, Laparoscopic hepatectomy, RCT

## Abstract

**Background:**

Laparoscopic hemihepatectomy (LH) has favorable short-term outcomes compared with open hemihepatectomy (OH), including shorter hospital stay. An in-depth healthcare utilization and cost-effectiveness analysis of the international multicenter ORANGE II PLUS randomized controlled trial comparing LH and OH was performed.

**Patients and Methods:**

Patients were randomly assigned to LH or OH in 16 European centers from October 2013 to January 2019. Costs were determined as a product of unit costs using patient-level, clinician-reported resource utilization up to 90 days. Item-specific resource use per country was presented. The measure of effect was quality-adjusted life year (QALY). Cost and effect differences were compared between treatment arms using nonparametric bootstrapping, from a Dutch healthcare cost perspective. A cost-effectiveness analysis was performed to establish the incremental cost-effectiveness ratio (ICER), i.e., costs per QALY gained, for LH compared with OH 1 year postoperatively.

**Results:**

Among 332 patients randomized to LH (*n* = 166) and OH (*n* = 166), intraoperative costs were higher for LH (LH 13,208 € versus OH 9437 €), while postoperative costs were lower for LH (LH 5774 € versus OH 7703 €). Longer operative time and greater instrument use contributed to higher intraoperative costs, while shorter hospital stays contributed to lower postoperative costs. Mean overall costs per patient were higher in LH (LH 18,982 € versus OH 17,141 €). The QALYs gained over 1 year postoperative were mean (standard deviation [SD]) 0.834 (0.218) for LH and mean 0.795 (0.237) for OH. The ICER was 36,677 € per additional QALY gained, and uncertainty analyses showed that LH had a 77% probability of being cost-effective compared with OH at a willingness-to-pay (WTP) threshold of 80,000 €.

**Conclusions:**

Although LH was more costly than OH, in a multicenter randomized trial, its clinical advantages translated into more QALYs gained over the first postoperative year and high probability of cost-effectiveness. These findings suggest that, where resources allow, LH may be preferred over OH for selected patients, offering both clinical benefits and acceptable economic value.

**Supplementary Information:**

The online version contains supplementary material available at 10.1245/s10434-025-18779-4.

Liver resection offers a curative option for patients with benign and malignant tumors of the liver and biliary tract. Minimally invasive surgery has recently been promoted as the preferred approach for hemihepatectomy in selected patients, as it is associated with quicker functional recovery and consequently shorter hospital stays without compromising oncological outcomes.^1–4^ Moreover, the lower physical impact of laparoscopic surgery has shown to significantly improve health-related quality of life (HRQoL) outcomes in a randomized controlled trial.^5^ Laparoscopic procedures do have their limitations, namely the higher technical complexity and demand for particular skills to manage bleeding, which results in longer operating times.^6^

Owing to longer operating times and more costly equipment, laparoscopic hemihepatectomy (LH) is a more expensive procedure than open hemihepatectomy (OH). Nonrandomized studies comparing open versus laparoscopic major liver resections have shown that higher operative costs of laparoscopy can be compensated by lower postoperative healthcare costs owing to favorable clinical outcomes.^7–12^ However, these conclusions are drawn from observational studies with a high risk of bias including small groups of patients. A randomized controlled trial comparing open and laparoscopic minor liver resections for colorectal liver metastasis found laparoscopy to be cost-effective.^13^ There is an urgent need to compare the costs of these two approaches for major liver resection, based on high-quality data from randomized trials. Demonstrating the cost-effectiveness of LH could further support the necessary investment in liver surgeons’ training to acquire the necessary skills to safely perform LH. It may also support the choice of laparoscopy in clinical decision-making regarding major liver resection and inform healthcare policymakers.

The ORANGE II PLUS international multicenter randomized controlled trial comparing LH and OH found that LH is associated with quicker functional recovery, shorter hospital stay, and improvements in quality of life.^4^ A concise summary of the cost-effectiveness analysis is also presented in the manuscript. However, comprehensive data are essential to inform policy decisions and facilitate extrapolation to other countries’ healthcare systems. This secondary study provides an in-depth insight into the postoperative healthcare-related resource use and quality of life over the first postoperative year. With these data, it estimates the cost-effectiveness of LH versus OH in terms of the incremental costs per quality-adjusted life year (QALY) gained.

## Patients and Methods

### Study Design, Setting, and Participants

The ORANGE II PLUS double-blind, randomized, controlled phase III trial was conducted in 16 centers across six European countries, specialized in hepatobiliary oncology. The methodology and design of the trial have been previously described.^4^ Eligible patients were adults who required a left or right hemihepatectomy (with or without the need for one additional hepatic wedge resection or metastasectomy) for accepted indications. Furthermore, they were only eligible with a body mass index (BMI) between 18 and 35 kg/m^2^ and an American Society of Anesthesiologists (ASA) physical status of I, II, or III. Treatment indication, feasibility, and safety of hemihepatectomy was assessed by a multidisciplinary team consisting of surgeons, radiologists, pathologists, hepatologists, oncologists, and radiotherapists. Patients were only approached for trial participation if the case was deemed appropriate for either of the surgical approaches. Patients were excluded from the trial when they previously had undergone any form of hepatectomy or were not eligible for a laparoscopic approach owing to insufficient margin from vascular or biliary structures. Pregnant or breastfeeding participants were also excluded.

### Randomization and Blinding

Patients were randomly assigned in a 1:1 ratio to either the OH or LH treatment arm. After obtaining written informed consent, patients were randomized using online randomization software (ALEA^®^) using a minimization scheme, stratified for hemihepatectomy side and treatment center.

Blinding for treatment allocation was applied. A large abdominal dressing covered all surgical incisions until day 4 after surgery to ensure patients and ward personnel were unaware of the allocated treatment (Appendix 1).

### Interventions

An Enhanced Recovery After Surgery (ERAS) Program was applied for all patients in the trial.^14^ The surgical intervention and the ERAS program were already part of current practice in the participating centers. The surgical approach was defined by randomization, while other choices regarding surgical technique, instrumentation, and methods were left to the surgeons’ discretion.

### Outcome Measures

#### Resource Use

A full overview of the unit costs and sources for each resource use component can be found in Table 1. Resource use was categorized into operative (anesthesia and surgical), postoperative, and out-of-hospital components. Resource use was collected up to 12 months. Resource use per country, and a detailed list of the most significant cost drivers, is presented to enable extrapolation to other countries.

**Table 1:** Resource-use items, prices, and cost information source

Resource-use item	Unit cost (range)^‖^	References
Anesthesia^*^		
Premedication	0.05–0.63	^1^
Epidural	32.74	^2^
Spinal/paravertebral anesthesia	15.02	^2^
Wound catheter	46.11	^2^
Infiltration with local anesthetic	1.53–3.60	^1^
Induction anesthetic	1.30–74.17	^1^
Maintenance anesthetic	3.03–643.57	^1^
Intravenous fluids, per liter	0.69–2.18	^2^
Transfusion	222.53	^3^
Perioperative antibiotics	5.29–19.32	^1^
Repeat of perioperative antibiotics	13.63	^1^
Antiemetics	0.32–4.80	^1^
Neuromuscular blockage relief	12.12	^1^
Perioperative inotropy	7.85–12.85	^1^
Disposable operative monitoring equipment^§^	0.61–201.61	^2^
*Surgical*		
Sitting time^†^, per minute	13.34	^2^
Cutting time^‡^, per minute	3.78	^2^
Pathological analysis	427.90	^4^
Operative disposables^§^		
Standard set open	69.44	^2^
Standard set laparoscopy	334.06	^2^
Open CUSA	419.91	^2^
Laparoscopic CUSA	497.86	^2^
Argon	101.17	^2^
Stapler	200.56	^2^
Open surgery vessel sealer	410.05	^2^
Laparoscopic vessel sealer	545.11	^2^
Specimen retrieval pouch	59.61	^2^
Trocar	27.80	^2^
Hemostatic sealants	21.39–366.00	^2^
*Postoperative*		
Hospital stay, per day	647.79	^3^
Laboratory blood sampling, per day	43.24	^3^
Time to oral analgesics, per day	56.85	^1^
Readmission (< 90 days), per day	641.99	^3^
Adverse event interventions		
Medical		
Antibiotics	228.94	^1^
Thrombotic event	89.05	^1^
Transfusion	222.53	^3^
CPR	677.75	^4^
C–D II not specified	233.25	^1^
Radiologic intervention		
US-guided drainage	325.26	^4^
CT-guided drainage	470.39	^4^
Endoscopic stent	1880.53	^4^
ERCP	1880.53	^4^
Coiling of vessel	3929.48	^4^
PTC drain	719.23	^4^
C–D III not specified	466.50	
Operation		
Local anesthesia	94.17	^4^
General anesthesia	5039.14	^4^
Intensive care		
Admission, per day	2033.17	^3^

*Medication doses were based on the highest recommended dose for each indication. For medications dosages based on weight, the average weight of the study population was used (75 kg). Medications dosed per unit of time were assumed to be administered for the average duration of the operation (254 min in the open group and 310 min for the laparoscopic group). For anesthetic gases, the average flow was assumed to be 2 L/min. Unless unavailable, costs for generic formulations were used

^†^Time between the patient entering and leaving the operating theatre. Includes: anesthesia and operating theatre staff wages, sterilization costs, postoperative recovery, general materials, building and energy expenses, supporting departments, other overhead expenses

^‡^Time between the start of incision and end of surgery. Includes: surgical staff wages and specific materials used by the surgical team

^§^Refer to Table 4

^¶^Costs are indicated per unit unless stated otherwise

^‖^Ranges are given for costs that differ depending on the specific medication or material used

^1^Medicijnkosten.nl^23^

^2^Local cost labels of the Maastricht University Medical Center (MUMC+)

^3^Dutch Guidelines for Execution of Economic Evaluations in Healthcare 2016 [Richtlijn voor het Uitvoeren van Economische Evaluaties in de Gezondheidszorg]^22^

^4^MUMC+ tariffs ledger per insurance declaration code^24^

*C–D* Clavien–Dindo grade, *CT* computed tomography, *ERCP* endoscopic retrograde cholangiopancreatography, *US* ultrasound, *PTC* percutaneous transhepatic cholangiogram, *SD* standard deviation, *CPR* cardiopulmonary resuscitation, *CUSA* Cavitron Ultrasonic Surgical Aspirator

#### Operative

Operative resource-use data were gathered by operating nurses who registered the use of all instruments and other items. The operative resource items were subdivided into anesthesia and surgical components. Anesthesia resource use included medication used, quantity of transfusions, infusion volume, and use of disposable materials for patient monitoring. Surgical resource use included operative time (sitting and cutting time) and disposable surgical material. Sitting time is defined as the interval between the arrival and departure of the patient from the operating room, and cutting time is defined as the period between the start of the incision and the closure of the skin.

#### Postoperative

Postoperative resource-use data were collected by clinicians and included a 90-day follow-up period following surgery. The following resource items were considered: length of stay, number of days on which laboratory blood sampling was performed, time to oral analgesia, interventions related to complications of Clavien–Dindo grade II or higher, and readmissions.^15^

#### Out-of-Hospital

Healthcare resource consumption of general practitioner (GP), emergency room (ER), and specialist visits following discharge, related to the operation, was gathered through patient-reported questionnaires at the follow-up moments at 3-, 6- and 12-months postoperative.^16^

### Costs

Costs were calculated as a product of the resource use quantity and unit costs of the respective resource use component. The cost analysis was performed from the perspective of the Dutch healthcare system. Cost prices were identified from multiple public and private sources (Table 1); the Dutch Guidelines for execution of Economic Evaluations in Healthcare 2016 (Richtlijn voor het Uitvoeren van Economische Evaluaties in de Gezondheidszorg), the Dutch federal medication costs website (medicijnkosten.nl), the Maastricht University Medical Centre tariffs ledger per insurance declaration code, and local cost labels at the Maastricht University Medical Center.^17–19^ Cost prices for surgical instruments were determined by averaging the costs of a specific instrument type from different suppliers. Costs were estimated on the value basis of 2016 since this was the year most resource-use data were collected. Given the relatively short follow-up (12 months), costs were not discounted. All costs are presented in euros (€) and, if retrieved from a difference source year, were adjusted for inflation on the basis of the Dutch Central Bureau of Statistics (CBS) yearly inflation rates.^20^

#### Operative

Micro-cost analysis was applied to determine differences in individual operative disposable items. Costs incurred during surgery were calculated on the basis of sitting and cutting time, abiding by the cost tariffs per minute used in the Maastricht University Medical Center. The sitting costs encompass the costs for the anesthesia and operating assistant personnel, sterilization, room costs, one night in postoperative recovery, energy expenditure, and taxes. Costs incurred by the surgical department staff are encompassed under the cutting costs. As the sitting or cutting time increases the duration of anesthesia, operative and overhead costs rise incrementally, as occurs in daily practice. Costs of medications, transfusions, infusions, and disposable materials were added to the sitting and cutting costs. Costs of reusable instruments were encompassed in the sterilization costs, while costs of additional disposable instrumentation were considered separately and added on. Aside from the instrumentation, a standard package of disposable materials was assumed for all cases that differed in price on the basis of the surgical approach. The contents of the standard package assumed for all cases in each treatment arm is found in Appendix 2.

Medication doses were estimated on the basis of the highest recommended dose for each indication.^21^ For medication dosages based on weight, the average weight of the study population was used (75 kg). Medications dosed per unit of time were assumed to be administered for the average duration of the operation stratified per treatment type. For anesthetic gases, the average flow was assumed to be 2 L/min. Unless unavailable, costs for generic formulations were used. Pathological analysis of surgical specimens was valued at a standard amount and was considered equal for all patients.

#### Postoperative

Hospital stay was valued at the standard rate per day in a university hospital. For costs of blood sampling analysis, a standard set consisting of 19 relevant laboratory measurements in the postoperative care of patients following liver surgery was assumed and valued at a standard price per day. The costs of postoperative pain medication were considered until the patient was switched to oral analgesics; thereafter, the costs were deemed negligible. Costs for hospital readmissions were valued at a standard rate per day. Costs for interventions related to complications were categorized into types of interventions, each with a standard cost. The costs of interventions for Clavien–Dindo grade I complications were considered negligible. Prolonged admission (> 1 day) to the high-dependency unit (HDU) postoperatively was also seen as an adverse-event intervention. The costs of the HDU were assumed to be equal to the costs of an intensive care unit (ICU) admission.

Costs for reintervention due to early recurrence or metastasis (within 90 days) were not considered as they were deemed to be independent of the surgical approach. No cost distinction was made between interventions for complications occurring during the initial hospital stay or following discharge. Costs associated with changes to prescription medication or prescription of additional medicines because of the operation were not considered.

### Health-Related Quality of Life (HRQoL)

The EQ-5D 3L™ questionnaire was assessed at baseline, discharge, and 10 days and 3-, 6-, and 12-months postoperatively to evaluate health-related quality of life (HRQoL) for the QALY estimation.^22^ This questionnaire uses five questions with three answers, ranging from no problems to severe problems, to describe a patient’s health state. Health states were transformed into utility scores (i.e., HRQoL scores) using the scoring algorithm proposed by Dolan et al. (1997) and based on each country’s value set.^23–28^ Since the Norwegian value set is currently lacking, the Swedish value set was used for patients treated in Norway.

### Cost-Effectiveness

A cost-effectiveness analysis was performed to support clinical decision-making regarding the cost per additional effect of introducing LH compared with OH. An empirical approach was applied without requiring model-based implementation. Effect (HRQoL) was measured with a follow-up of 12 months, and costs were based on clinician-reported 90-day follow-up data, assuming equal costs between interventions after this time point. The outcome measure of effect is the QALY. Cost-effectiveness is expressed in the incremental cost-effectiveness ratio (ICER):

$${\mathrm{ICER}} = \, \left( {{\text{cost of LH }} - {\text{ cost of OH}}} \right){\text{ divided by }}\left( {{\text{effect of LH }} - {\text{ effect of OH}}} \right) \, = \, \Delta {\text{ costs}}:\Delta {\text{ effects}}$$

If the ICER falls below the willingness-to-pay (WTP) threshold, the intervention is considered cost-effective. In the Netherlands, a threshold of up to 80,000 € per QALY gained is used in conditions with a high disease burden.^29–32^

### Sample Size

A drop-out rate of 10% and a loss in degrees of freedom for estimating covariate effects (hemihepatectomy side and center) were anticipated, leading to a total sample size of 250 patients to demonstrate a 2-day reduction in time to functional recovery, i.e., the primary outcome of the trial, with a two-sided 4% level of significance and a power of 80%, assuming a standard deviation (SD) of time to functional recovery of 5 days within both groups. A value of 4% was used to correct for bias caused by interim analysis. On the basis of the interim analysis, with an effect size being lower than expected, the sample size was extended to 350 patients. Only the primary outcome was assessed during interim analysis and did not influence healthcare utilization, costs, and cost-effectiveness outcomes or analysis.

### Statistical Analysis

This study followed the CONSORT 2025 expanded checklist found in Appendix 3. To account for missing data on operative disposables, the average expenditure per operation was calculated and stratified by treatment type. This average cost per treatment type was then applied to patients with missing data. For patients with missing data on cutting time, the average time for the rest of the study population, stratified per treatment type, was assumed. For missing sitting time, the average difference between sitting and cutting time was stratified per treatment type and added onto the cutting time. The cost-questionnaire data were evaluated for completeness for each follow-up time point. Owing to low response rates of the cost questionnaires and intercountry heterogeneity owing to local protocols and arrangements of healthcare systems, only the 90-day clinician-reported outcomes were included as cost drivers in the cost analysis, except for readmission length. Multiple imputation was applied for missing values of the EQ-5D 3L™.^33^ Five imputed datasets were generated. QALY’s were estimated on the basis of these five datasets and pooled into one dataset using Rubin’s rule, which was then used in the cost-effectiveness analysis.^34^

Continuous data were expressed as means with standard deviations (SD), discrete data as medians with interquartile ranges (IQR), and categorical variables as absolute numbers and percentages, unless indicated otherwise. Where means are reported, *t*-tests were used, otherwise Mann–Whitney *U* test was applied. Categorical variables were analyzed using the chi-squared or Fisher’s exact tests where appropriate.

QALYs were calculated by determining the area under the curve using the average utility score between two follow-up time points and multiplying this by the time between these time points as a fraction of a year. In case of death, the EQ-5D 3L™ utility score was assumed to be zero. Cost and QALY differences between the groups were estimated using nonparametric bootstrapping with 1000 repetitions and are reported as bootstrapped mean and bootstrapped 95% confidence intervals (BCIs). To address uncertainty around the ICER, nonparametric bootstrapping with 5000 repetitions was used. The bootstrap estimates of the joint mean cost and mean effect differences was plotted in a cost-effectiveness analysis plane (CEAP) to visualize their distribution. A cost-effect pair located in the northeast quadrant, for example, indicates that the LH is on average more effective and costly than OH, while a cost-effect pair located in the southeast quadrant indicates that the LH is on average more effective and less costly (dominant) than the OH. Finally, a cost-effectiveness acceptability curve (CEAC) was constructed to estimate the probability of cost-effectiveness for various maximum WTP thresholds.^35^ A sensitivity analysis was performed in which the Dutch value set was applied to the EQ-5D 3L health states of all patients. A subgroup analysis of the laparoscopic procedures was performed comparing the total costs of the first and second half of procedures per center.

Statistical significance was defined as a *p* < 0.05 or 95% BCI of mean differences not including zero. Analyses were performed according to modified intention-to-treat, excluding patients who did not undergo surgery after randomization and analyzing patients in the group they were randomized to if they did undergo surgery (i.e., conversions were analyzed in the LH group). All analyses were carried out using IBM SPSS Statistics software, version 26.0 (IBM Corp., Armonk, New York, USA), Excel for bootstrapping, and R statistical computing for Windows version 4.1.0. The results are reported according to the Consolidated Health Economic Evaluation Reporting Standards (CHEERS) statement (Appendix 4).^36^

### Ethics Approval

Ethical approval of the study protocol was obtained from Maastricht University Medical Center (METC NL36215.068.11). The study was designed by the authors and is registered at ClinicalTrials.gov (NCT01441856). All patients were given a detailed description of the study, including contact information of the researcher at least 1 week prior to inclusion. Written informed consent was obtained from all participating patients. Patients received no financial compensation. The study received no commercial funding. Anonymity and confidentiality were guaranteed for the patients regarding the obtained data. The trial was conducted in accordance with the Declaration of Helsinki and with Good Clinical Practice as defined by the International Conference of Harmonization.

## Results

### Characteristics of the Included Participants

**Fig. 1:**
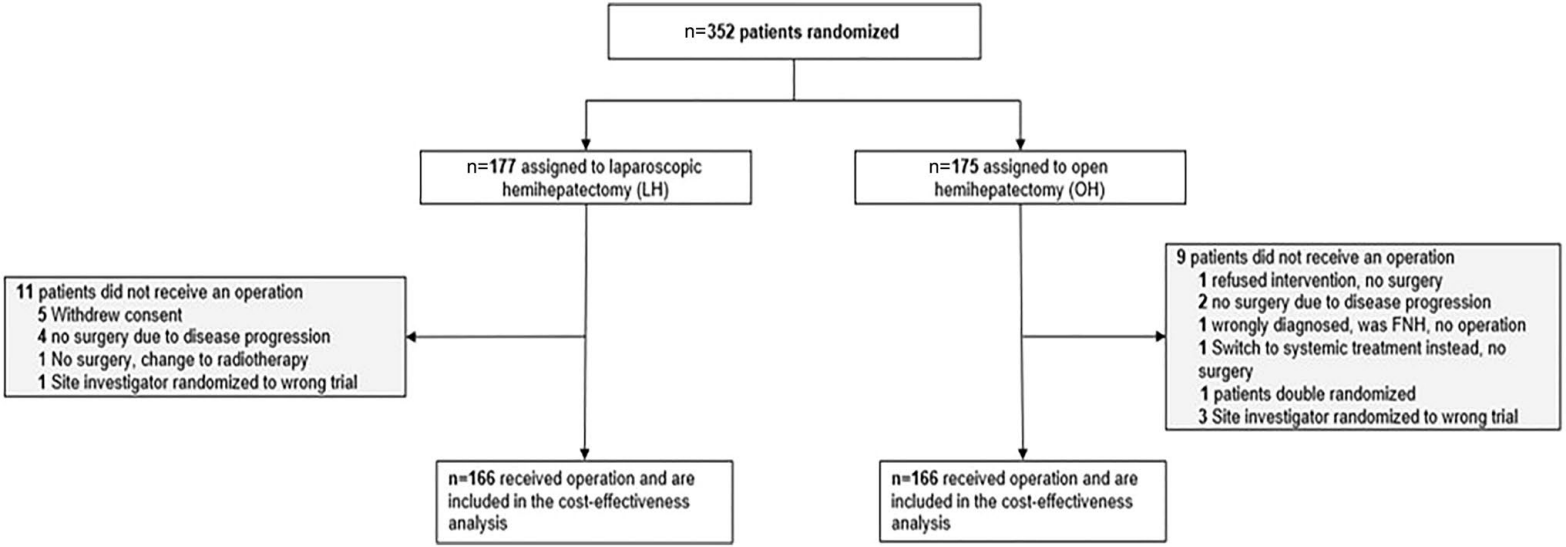
Study CONSORT flow diagram

Between October 2013 and January 2019, 352 patients were randomized into LH (*n* = 177) and OH (*n* = 175) (Fig. 1). After excluding dropouts, 332 patients proceeded to surgery by either laparoscopy (*n* = 166) or open surgery (*n* = 166) and were included in the modified intention-to-treat analysis. Table 2 reports the baseline characteristics of the patients in both groups. Colorectal liver metastases were the most common indication for surgery (59.1% of malignant cases).

**Table 2:** Baseline characteristics

Characteristic	OH (*n* = 166)	LH (*n* = 166)
Male, *n* (%)	96 (57.8)	99 (59.6)
Age		
years, mean (SD)	62.6 (13.0)	61.5 (13.5)
≥ 65 years, *n* (%)	87 (52.4)	77 (46.4)
BMI, kg/m^2^, median (IQR)	25.0 (22.0–28.0)	26.0 (23.0–29.0)
ASA classification, *n* (%)		
I: Healthy	19 (11.4)	13 (7.8)
II: Mild systemic disease	91 (54.8)	93 (56.0)
III: Severe systemic disease	52 (31.3)	52 (31.3)
ECOG performance status score, *n* (%)		
0: Asymptomatic, normal activity	123 (74.1)	121 (72.9)
1: Symptomatic, normal activity	40 (24.1)	36 (21.7)
2: Symptomatic, < 50% bedridden	1 (0.6)	4 (2.4)
3: Symptomatic, > 50% bedridden	0	1 (0.6)
4: 100% bedridden		
Charlson Comorbidity Index, mean (SD)	6.18 (2.8)	6.33 (3.2)
Previous abdominal surgery, *n* (%)	92 (55.4)	87 (52.4)
Preoperative portal vein embolization, *n* (%)	9 (5.4)	16 (9.6)
Preoperative chemotherapy, *n*/total *N* (%)	61/145 (42.1)	53/136 (39.0)
Radiological diagnosis, *n* (%)		
Benign	20 (12.3)	30 (18.3)
Hemangioma	6 (3.6)	6 (3.6)
Adenoma	0	5 (3.0)
Follicular nodular hyperplasia	2 (1.2)	0
Other benign	12 (7.2)	15 (9.0)
Malignant	145 (87.3)	136 (81.9)
Colorectal metastasis	79 (47.6)	89 (53.6)
Hepatocellular carcinoma	25 (15.1)	22 (13.3)
Cholangiocarcinoma	30 (18.1)	17 (10.2)
Other malignant	12 (7.2)	11 (6.6)
Country, *n* (%)		
The Netherlands	20 (12.0)	20 (12.0)
Germany	4 (2.4)	3 (1.8)
Belgium	36 (21.7)	36 (21.7)
England	56 (33.7)	59 (35.5)
Italy	44 (26.5)	41 (24.7)
Norway	6 (3.6)	7 (4.2)
Hemihepatectomy side, *n* (%)		
Left	58 (34.9)	61 (36.7)
Right	108 (65.1)	105 (63.3)
Additional contralateral surgery, *n* (%)		
Wedge resection	18 (10.4)	18 (10.1)
Ablation	3 (1.7)	6 (3.4)
Ablation and wedge resection	2 (1.2)	2 (1.1)

*ASA* American Society of Anesthesiologists, *BMI* body mass index, *ECOG* Eastern Cooperative Oncology Group, *IQR* interquartile range, *LH* laparoscopic hemihepatectomy, *OH* open hemihepatectomy

### Resource Use

Resource use is presented in Tables 3 and 4. Country-specific resource use is found in Appendix 5. LH had higher mean cutting times (316 min versus 251 min, *p* < 0.001) and sitting times (417 min versus 358 min, *p* < 0.001) compared with OH. The higher mean sitting and cutting time for laparoscopy was apparent across all participating countries (Appendix 6). More staplers were used in LH than in OH (477 units versus 279 units). In addition, electrothermal tissue sealers were also used more often in LH (110 units versus 9 units). A median of 5 (IQR 4–6) trocars were used per laparoscopic case, while, obviously, this instrument was not required in open procedures. Patients undergoing LH had a shorter median length of stay (5 days versus 6 days, *p* = 0.001) and shorter median use of non-oral analgesia (2 days versus 3 days, *p* < 0.001). In total, 133 (40%) patients received an intervention as a result of an adverse event (Clavien–Dindo ≥ II), this was similar across the two groups (42% OH versus 39% LH, *p* = 0.58). Clinician reported readmission at 90 days postoperatively was similar between OH and LH (13% versus 14%, *p* = 0.87).

**Table 3:** Intraoperative and postoperative resource utilization, pooled

	OH (*n* = 166)	LH (*n* = 166)	*p*-Value
*Intraoperative*			
Transfusion requirement, units, mean (SD)	0.27 (1.81)	0.24 (0.72)	0.848
Cutting time, minutes, mean (SD)	*n* = 162	*n* = 162	
	251 (83)	316 (95)	< 0.001
Sitting time, minutes, mean (SD)	*n* = 137	*n* = 130	
	358 (83)	417 (95)	< 0.001
Operative disposables	Displayed in Table 5		
*Postoperative*			
Length of stay, days, median (IQR)	*n* = 166	*n* = 166	
	6 (5–7)	5 (4–7)	0.001
Time to oral analgesics, days, median (IQR)	*n* = 158	*n* = 163	
	3 (5–7)	2 (4–7)	< 0.001
Blood sampling, days, median (IQR)	*n* = 166	*n* = 166	
	4 (3–4)	4 (3–4)	0.213
90-day readmission			
Readmission, *n* (%)	*n* = 152 20 (13)	*n* = 156 22 (14)	0.869
Readmission length*, days, median (IQR)	*n* = 13 7 (5–13)	*n* = 15 5 (3–9)	
Adverse event interventions at 90 days, *n* (%)	*n* = 166	*n* = 166	
	69 (42)	64 (39)	0.575
Medical Antibiotic courses Thrombotic events Transfusions CPR C–D II not specified	42 3 14 1 1	29 3 8 0 3	
Radiologic intervention US-guided drainage CT-guided drainage Endoscopic stent ERCP Coiling of vessel PTC drain C–D III not specified	17 0 1 1 0 0 2	12 2 0 5 1 1 4	
Operation			
Under local anesthesia	1	1	
Under general anesthesia	8	4	
Intensive care	41	32	0.233
Admission, admission length, total days	127	45	0.019
Healthcare consults^*^			
GP, mean (SD)/total			
3 months	*n* = 109	*n* = 106	
	1.5 (2.2)/103	0.9 (1.4)/99	
6 months	*n* = 97	*n* = 92	
	0.9 (1.8)/84	0.7 (1.6)/66	
12 months	*n* = 81	*n* = 90	
	0.3 (0.8)/24	0.5 (1.3)/47	
ER, mean (SD)/total			
3 months	*n* = 110	*n* = 110	
	0.2 (0.6)/19	0.1 (0.4)/15	
6 months	*n* = 99	*n* = 98	
	0.1 (0.6)/12	0.1 (0.4)/9	
12 months	*n* = 81	*n* = 75	
	0.0 (0.1)/1	0.0 (0.3)/4	
Specialist, mean (SD)/total			
3 months	*n* = 111	*n* = 111	
	1.5 (1.9)/161	1.6 (1.8)/174	
6 months	*n* = 100	*n* = 99	
	1.2 (2.5)/119	1.1 (1.6)/109	
12 months	*n* = 81	*n* = 91	
	1.1 (1.7)/87	1.1 (1.9)/99	

*Patient-reported outcome

Units are total quantities unless stated otherwise

*C–D* Clavien–Dindo grade, *CT* computed tomography, *ERCP* endoscopic retrograde cholangiopancreatography, *IQR* interquartile range, *LH* laparoscopic hemihepatectomy, *OH* open hemihepatectomy, *SD* standard deviation, *US* ultrasound, *PTC* percutaneous transhepatic cholangiogram

**Table 4:** Resource utilization of operative disposables, pooled

Operative disposables*	OH (*n* = 166)	LH (*n* = 166)
*Anesthesia*		
	*n* = 163	*n* = 161
Epidural, *n* (%)	67 (40)	53 (32)
Spinal/paravertebral anesthesia, *n* (%)	15 (9)	19 (11)
Wound catheters, *n*	33	7
Arterial line, *n*	162	158
Central venous line, *n*	122	120
*Surgical*		
	*n* = 108	*n* = 92
CUSA	98	8
Laparoscopic CUSA	0	75
Argon	56	29
Staplers	279	477
Electrothermal tissue sealers	9	110
Trocars	0	459
	*n* = 156	*n* = 146
Hemostatic sealant	145	119

*Not all operative disposables included in the cost calculation are displayed. Smaller cost drivers such as sutures and drains were also considered in the micro-costing analysis

Units are total quantities unless stated otherwise

*CUSA* Cavitron Ultrasonic Surgical Aspirator, *LH* laparoscopic hemihepatectomy, *OH* open hemihepatectomy

Response rates to the patient-reported resource use questionnaires at 10 days and 3-, 6- and 12-months follow-up were 86%, 67%, 60%, and 52%, respectively. The response rates were similar in both treatment groups. Only 44% of patients responded to all follow-up questionnaires. The number of patients requiring consultations with the GP, ER, and specialists showed a decreasing trend over the course of the first postoperative year. This trend was also observed for the mean number of visits. No apparent differences in patient-reported healthcare resource use were observed between LH and OH across all follow-up time points. Small intercountry differences were observed in resource use utilization of GP, ER, and specialist consultations (Appendix 5).

### Costs

**Fig. 2:**
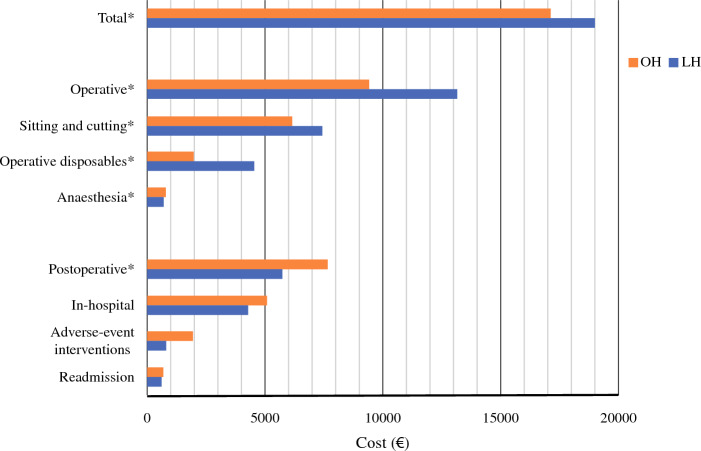
Cost distribution over 90 days postoperatively. ^*^Statistically significant bootstrapped mean difference. OH, open hemihepatectomy, LH laparoscopic hemihepatectomy

Mean costs per patient are presented in Table 5 and Fig. 2. Total 90-day costs per patient were higher in LH 18,982 € (95% BCI 17,966–20,199 €) than OH 17,141 € (95% BCI 15,729–18,700 €), with a statistically significant mean difference of 1841 € (95% BCI 108–3692 €). Operative costs contributed to 55% of the costs in OH and 70% in LH (Fig. 2). Operative costs were mean 13,208 € (95% BCI 12,738–13,702 €) for LH versus 9437 € (95% BCI 9069–9879 €) for OH, with a statistically significant mean difference of 3771 € (95% BCI 3102–4370 €), resulting in a mean difference of 1309 € (95% BCI 998–1648 €). Data from 200 patients (61%; Table 4) were available for micro-cost analysis. Higher costs incurred per case through use of operative disposables also contributed to higher operative costs in LH. On average, 2547 € (95% BCI 2257–2883 €) more was spent per case on operative disposables in the laparoscopic group.

**Table 5:** Overview of mean operative and postoperative 90-day healthcare costs

	Open hemihepatectomy	Laparoscopic hemihepatectomy	Cost difference
*Operative*			
Anesthesia	803 (536–1177)	717 (557–990)	− 86 (− 316 to 520)
Sitting and cutting	6169 (5938–6413)	7478 (7244–7744)	1309 (998–1648)
Operative disposables^*^	2037 (1902–2171)	4584 (4323–4880)	2547 (2257–2883)
*Postoperative*			
In-hospital admission^†^	5049 (4418–5683)	4307 (3822–4912)	− 742 (− 1563 to 134)
Readmission	704 (367–1106)	622 (290–1071)	− 82 (− 526 to 603)
Adverse event interventions	1950 (1140–3165)	844 (594–1120)	− 1106 (− 2318 to 264)
*Operative costs*^*‡*^*:*	9437 (9069–9879)	13,208 (12,738–13,702)	3771 (3102–4370)
*Postoperative costs*^*§*^*:*	7703 (6478–9096)	5774 (4999–6679)	− 1929 (− 3596 to − 361)
*Total costs:*	17,141 (15,729–18,700)	18,982 (17,966–20,199)	1841 (108–3692)

Values are means (nonparametric bootstrapped 95% confidence intervals) given in euros and adjusted for inflation to the year 2016

*Calculated from available data from 201 patients (109 open and 92 laparoscopy)

^†^Length of stay, blood sampling, and time to oral analgesics

^‡^Operative disposables, anesthesia medication, anesthesia disposables, sitting time, cutting time, and pathological specimen analysis

^§^Length of stay, time to oral analgesia, blood sampling, readmission, and adverse event interventions

The postoperative costs for LH and OH were 5774 € (95% BCI 4999–6679 €) and 7703 € (95% BCI 6478–9096 €) respectively; mean difference −1929 € (95% BCI −3596 to 361 €). Bootstrapped mean cost differences for the in-hospital stay did not differ significantly between the groups −742 € (95% CI −1563 to 134 €). Costs incurred for 90-day readmission and interventions due to adverse events did not differ significantly between LH and OH.

A subgroup analysis of laparoscopic procedures in the first and second half of inclusions per center found no significant differences in costs between these two periods (early 19,295 € [95% CI 17,707–20,882 €] versus late 18,655 € [95% CI 17,121–20,187 €]; mean difference −640 € [95% BCI −2712 to 1696 €]).

### HRQoL

The observed and imputed HRQoL outcomes, as measured by the EQ-5D 3L™, are reported in Tables 6, respectively. The imputed set had lower utility scores. EQ-5D 3L™ utility scores at baseline were nearly identical for the two treatment arms, and thus, did not necessitate correction. The distribution of missing data was similar across groups. The QALYs gained over 1 year postoperative were a mean of 0.834 (SD 0.218) for LH and a mean of 0.795 (SD 0.237) for OH, with a mean difference of 0.039 (95% BCI − 0.025 to 0.103). HRQoL outcomes per country are presented in Appendix 5.

**Table 6:** Mean EQ-5D 3L utility scores per time point in observed and imputed data sets

Follow-up time point	Observed		Imputed	
	OH (*n* = 166)	LH (*n* = 166)	OH (*n* = 166)	LH (*n* = 166)
Baseline	*n* = 165	*n* = 164		
	0.849 (0.228)	0.851 (0.210)	0.852 (0.225)	0.856 (0.207)
Discharge	*n* = 133	*n* = 142		
	0.586 (0.298)	0.680 (0.257)	0.574 (0.301)	0.667 (0.263)
10-day follow-up	*n* = 132	*n* = 136		
	0.669 (0.293)	0.754 (0.247)	0.651 (0.299)	0.740 (0.247)
3-month follow-up	*n* = 128	*n* = 131		
	0.811 (0.253)	0.854 (0.228)	0.770 (0.290)	0.824 (0.252)
6-month follow-up	*n* = 124	*n* = 124		
	0.796 (0.291)	0.831 (0.238)	0.752 (0.315)	0.783 (0.275)
12-month follow-up	*n* = 113	*n* = 119		
	0.746 (0.356)	0.792 (0.321)	0.711 (0.342)	0.766 (0.306)
QALYs	*n* = 83	*n* = 90		
	0.795 (0.237)	0.834 (0.218)	0.730	0.780

Values are mean (SD)

*LH* laparoscopic hemihepatectomy, *OH* open hemihepatectomy

A greater proportion of patients in the OH group reported problems (some or extreme problems) at discharge in the self-care, pain/discomfort, and anxiety/depression dimensions of the EQ-5D 3L (Table 7). At 10-day follow-up, patients who had undergone OH had problems in the mobility, self-care, and pain/discomfort dimensions more frequently compared with LH. In addition, at 3 months, there were more problems in the mobility, usual activities, and pain/discomfort dimensions in the OH group.

**Table 7:** Patients reporting any problems on the EQ-5D 3L per dimension, per time point

Follow-up time point	Dimension	Patients reporting any problems, *n* (%)	
		LH (*n* = 166)	OH (*n* = 166)
Baseline	Mobility	17 (10)	24 (15)
	Self-care	7 (4.2)	10 (6.1)
	Usual activities	31 (19)	31 (19)
	Pain/discomfort	44 (27)	45 (27)
	Anxiety/depression	49 (30)	46 (28)
Discharge	Mobility	82 (49)	94 (57)
	Self-care	55 (33)	81 (49)
	Usual activities	128 (77)	135 (81)
	Pain/discomfort	103 (63)	125 (76)
	Anxiety/depression	52 (31)	71 (43)
10 days	Mobility	56 (33)	86 (52)
	Self-care	37 (23)	53 (32)
	Usual activities	106 (64)	114 (69)
	Pain/discomfort	88 (54)	106 (64)
	Anxiety/depression	45 (27)	56 (34)
3 months	Mobility	35 (21)	50 (30)
	Self-care	14 (8.2)	22 (13)
	Usual activities	51 (31)	68 (41)
	Pain/discomfort	57 (35)	74 (45)
	Anxiety/depression	48 (30)	49 (30)
6 months	Mobility	44 (26)	55 (33)
	Self-care	22 (13)	31 (19)
	Usual activities	51 (31)	57 (34)
	Pain/discomfort	55 (34)	62 (37)
	Anxiety/depression	58 (35)	48 (29)
12 months	Mobility	42 (25)	49 (29)
	Self-care	26 (15)	30 (18)
	Usual activities	50 (30)	64 (39)
	Pain/discomfort	53 (32)	66 (40)

### Cost-Effectiveness

**Fig. 3:**
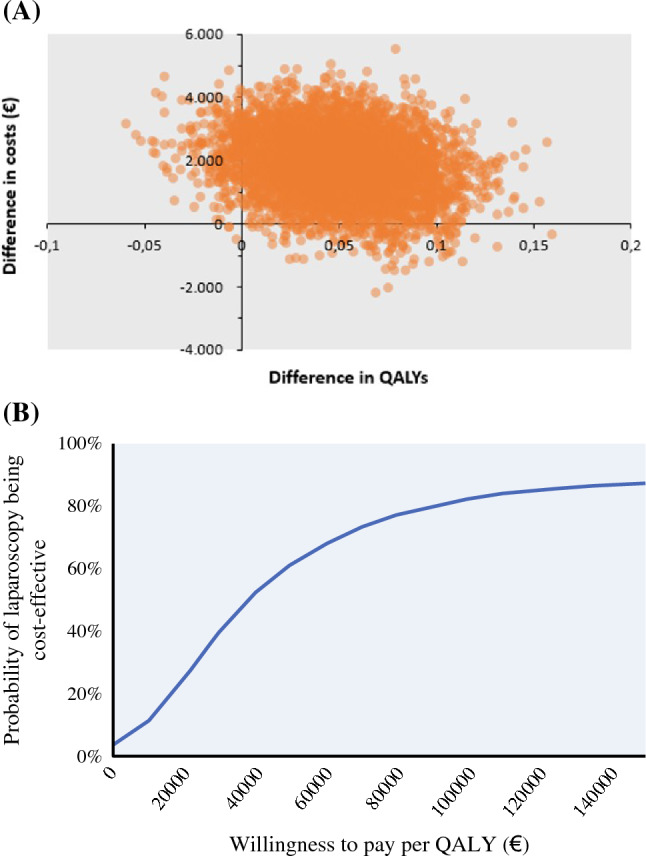
**a** Cost-effectiveness analysis plane. Cost effectiveness plane for 5000 bootstrapped repetitions showing differences in total costs and QALYs between open and laparoscopic hemihepatectomy. A majority of the datapoints lie in the right upper quadrant, demonstrating laparoscopic hemihepatectomy is more effective but more costly. **b** Cost-effectiveness analysis probability curve. Cost-effectiveness acceptability curve showing the probability of laparoscopic hemihepatectomy being more cost-effective for various willingness-to-pay thresholds per QALY

The probability of LH being cost-effective was 77% for a maximum WTP threshold of 80,000 € (ICER = 36,677 €) compared with OH owing to its higher QALYs gained. Figure 3a shows the differences in costs and QALYs between LH and OH in the 5000 bootstrapped repetitions. The distribution of the data points lies primarily in the right upper quadrant (92%), indicating higher effectiveness of LH in terms of QALYs gained; however, it is accompanied by higher costs. Figure 3b shows the probability of LH being more cost-effective than OH for various WTP thresholds per QALY.

A sensitivity analysis applying the Dutch value set to the EQ-5D 3L scores showed similar results (ICER = 41,204 €) (Appendix 6). The probability of LH being cost-effective was 71% for a maximum WTP of 80,000 €.

## Discussion

This healthcare utilization, costs, and cost-effectiveness analysis, from a healthcare perspective, with data from the ORANGE II PLUS international multicenter randomized controlled trial in laparoscopic and open hemihepatectomy across 16 high-volume liver surgery centers in Europe, displays a comprehensive overview of resource use per country, demonstrating higher operative times in LH as well as higher use of staplers and electrothermal tissue sealers as compared with OH. Cost-effectiveness analysis revealed that although overall costs of LH were higher than OH, LH had a higher gain in QALYs over the first year after resection. This resulted in an ICER of 36,677 € and a 77% probability that LH is cost-effective compared with OH at a maximum WTP threshold of 80.000 €.

Investments in new surgical interventions require thorough evaluation, not only of safety and clinical effectiveness, but also regarding the impact on healthcare resource use, costs, and cost-effectiveness. This assessment involves an analysis of the direct costs of implementation and also weighs the potential benefits in terms of patient outcomes. Comprehensive assessment of these factors in policymaking ensures that healthcare resources are allocated efficiently while maximizing patient benefit.

At 90 days, the higher intraoperative costs of LH were not fully compensated by its lower postoperative costs, ultimately resulting in overall higher total costs compared with OH. LH incurred higher costs intraoperatively, which can be explained by the longer sitting and cutting times accompanied by higher sitting and cutting costs compared with OH. This economic disadvantage may diminish with greater surgeon and center experience. At the start of the study period, most participating centers were still within their learning curve. As experience with LH increases, operating times could decrease, leading to a corresponding reduction in costs.^37,38^ In the present study, total costs per patient in the first and second halves of inclusions per center did not differ significantly. However, the study period was relatively short, and most centers included fewer than ten patients undergoing LH. Consequently, potential cost-reduction effects through gained experience may not yet have become evident. Given the long learning curve associated with major liver resections, greater surgical experience may be required before meaningful cost reductions can be achieved.^39^ In addition, the greater use of expensive disposable instrumentation such as electrothermal vessel sealers, staplers, and trocars in LH further contributed to higher intraoperative costs. Implementing reusable tools in laparoscopic surgery has the potential to yield significant cost savings, as well as reduce its carbon footprint.^40–42^

To the best of our knowledge, this is the first cost-effectiveness analysis comparing laparoscopic and open major liver resection in a randomized controlled trial. The results from our study are consistent with those of the OSLO-COMET trial, which compared the cost-effectiveness of laparoscopic versus open minor liver resections for colorectal liver metastases.^13^ Fretland and colleagues found that laparoscopy was cost-effective compared with open surgery, at a likelihood of 67% for a maximum WTP of US $95,000. Similar to the present study, previous retrospective cost-analyses of major liver resection have reported higher intraoperative costs for laparoscopy.^7–12,43^ However, in contrast to the present study, these previous reports found that higher intraoperative costs for laparoscopic procedures were eventually offset by lower postoperative costs owing to shorter hospital stays and lower complication rates than open liver resection. The difference between our findings and those of previous studies is likely due to the smaller gap in length of hospital stay observed in the present trial—just 1 day between laparoscopy and open surgery—compared with the 2–6-day difference reported in retrospective studies. Potentially, the implementation of an ERAS™ program in the current study optimized recovery following both surgical procedures, thereby minimizing the postoperative differences between LH and OH. Moreover, these retrospective studies were naturally prone to selection bias.

Quality-of-life analyses in the present study indicated that LH was associated with a gain of 0.039 QALYs over a 1-year period compared with OH, corresponding to approximately 14 additional days in perfect health. In this study, the difference in QALYs is a result of the reduced physiological burden and faster recovery associated with the laparoscopic approach, primarily during the short-term postoperative phase rather than survival benefits. These higher quality-of-life measures were maintained throughout the first postoperative year. Consistent with these findings, a previous quality-of-life analysis from the ORANGE II PLUS trial reported clinically relevant improvements in multiple quality-of-life domains favoring the laparoscopic technique.^44^ Importantly, the QALY difference in the presented study translated into an ICER of approximately 36,000 € per QALY, which would generally be considered acceptable and a value for money in many high-income settings, whereas affordability and resource availability are more critical considerations in low- and middle-income contexts.^45^ Hence, the decision to implement the laparoscopic technique is dependent on its specific health system context and maximum WTP. The present study focused on healthcare-related costs. However, implications of these approaches on other factors such as societal costs may influence the overall costs per patient. The reduced physical impact of laparoscopic surgery could continue to benefit patients in their home setting, potentially leading to a faster return to work and, consequently, lower societal costs. In this study, over half (51%) of the patients were under 65 years, the retirement age in the Netherlands at that time, and could be considered active contributors to the workforce. Unfortunately, a high volume of missing data on patient-reported outcomes such as return to work and need for temporary care at home prevented reliable analysis of potential societal cost savings.

A relative limitation of the current study is that the costs are presented from the perspective of the Dutch healthcare system (i.e., Dutch cost prices were applied to the resource use of all patients), a choice made for pragmatic reasons. Moreover, the maximum WTP is a subjective value with variations between countries, meaning that the cost-effectiveness conclusions drawn in one country may not be directly applicable to another. To ensure generalizability to other countries, we have presented resource use in detail. Similar resource utilization was observed across the participating countries, and we do not expect this to significantly impact the overall results. Although formal scenario analyses using cost structures from other countries were not conducted, proportional differences in unit costs can be expected to result in comparable proportional changes in total costs and, assuming QALYs remain constant, in the ICER. Furthermore, differences in the relative prices of key cost drivers, such as hospital admissions and operating room utilization, could also meaningfully affect the ICER if these relative price structures vary considerably between countries. By presenting our resource use data transparently, we enable researchers and policymakers to adapt the cost analysis to their own national contexts and to estimate context-specific cost-effectiveness outcomes.

Costs for reintervention due to early recurrence or metastasis (within 90 days) were not considered in this trial as they were deemed to be independent of the surgical approach. This is supported by current data with seven patients having disease recurrence within 90 days in both groups, and two patients starting chemotherapy as treatment for recurrence before 90 days in both groups. Another limitation is that costs were estimated on the basis of 2016 values. While this provides an accurate reflection of cost-effectiveness during the trial period, current costs may differ owing to inflation and changes over time. It is also conceivable that the costs of laparoscopic surgery have decreased as the technique has become more widely adopted and production processes have become more standardized.

While we aimed to provide a comprehensive overview of the cost expenditure for both LH and OH, a balance had to be struck between practicality and accuracy. Therefore, only the main cost drivers were considered. As a result, the costs calculated in this study may underestimate the actual costs incurred in practice owing to the missing inclusion of small expenditures. However, we do not anticipate significant differences in the smaller cost drivers between the two treatment arms. In addition, large amounts of missing data for patient-reported outcomes—such as the number of visits to the GP, ER, and specialists—led to the omission of detailed information on these variables to preserve the reliability of the analysis. Hence, the costs presented only include hospital-related costs up to 90 days. We acknowledge that excluding patient-reported healthcare use and broader societal costs may lead to a potential underestimation of the broader economic advantages of LH, particularly if patients return to normal activities sooner and require fewer community-based health services such as a general practitioner, physiotherapy, and homecare. We do not anticipate major differences between LH and OH beyond the 90-day time point, or in oncologic outcomes. Available patient-reported resource use and HRQoL data within the first year demonstrate the greatest advantages for LH within the first 3 months. However, certain long-term complications such as incisional hernias or complications related to abdominal adhesions could potentially favor the cost-effectiveness of the laparoscopic approach.

## Conclusions

Our study offers valuable insights into country-specific and item-specific resource use, as well as the cost-effectiveness of LH compared with OH, providing an essential foundation for guiding healthcare resource allocation. The findings support the broader implementation of LH in clinical practice, demonstrating that the adoption of LH represents an investment into patients’ quality of life within socially acceptable financial margins. Healthcare systems should be prepared to make economic investments to improve implementation and surgical training in minimally invasive liver resections to increase the accessibility of laparoscopic major hepatectomy and improve patients’ quality of life.

## Supplementary Information

Below is the link to the electronic supplementary material.

Supplementary file1 (DOCX 554 KB)

## Data Availability

Data collected for the study, including deidentified individual participant data and a data dictionary defining each field in the set, can be made available to others on reasonable request and after signing appropriate data sharing agreements after all following studies on this main paper by the research team have been concluded. Please send data access requests to r.van.dam@mumc.nl. Such requests must be approved by the respective ethics boards and appropriate data custodians.
