# Supplementary material for: Healthcare Utilization, Costs, and Cost-Effectiveness of Patients Undergoing Laparoscopic and Open Hemihepatectomy: A Secondary Analysis of the ORANGE II PLUS Randomized Controlled, Phase 3, Superiority Trial
Source: Ann Surg Oncol. 2025 Dec 12;33(4):2882–98. doi: 10.1245/s10434-025-18779-4 (PMC12982277; doi:10.1245/s10434-025-18779-4)
Supplement: Supplementary file 1 — Supplementary file1 (DOCX 554 KB) [file 10434_2025_18779_MOESM1_ESM.docx]

# **Appendix**

APPENDIX 1: Abdominal dressing for patient blinding

**
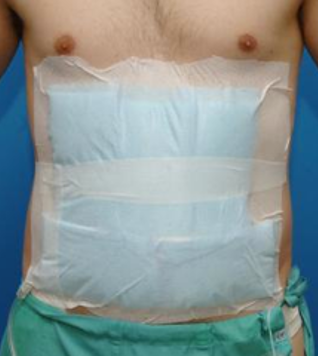
**

APPENDIX 2: Contents standard package operative disposables

| **Open hemihepatectomy** |
| --- |
| Sterile covers (operation field, instrument field) |
| 3M ™ Ioban ™ |
| Sterile gowns |
| 30x Gauzes and 3x abdominal compresses |
| Scalpel |
| Diathermia |
| Irrigation and suction set |
| **Laparoscopic hemihapatectomy** |
| Sterile covers (operation field, instrument field, laparoscopic scope) |
| 3M ™ Ioban ™ |
| Sterile gowns |
| 30x Gauzes and 3x abdominal compresses |
| Insufflation cord |
| Syringe |
| Scalpel |
| Diathermia |
| Laparoscopic irrigation and suction set |
| Laparoscopic graspers, bipolar forceps and scissors |

APPENDIX 3: CONSORT 2025 expanded checklist of detailed information to include when reporting a randomised trial

|  | Section/topic | No | CONSORT 2025 checklist item description | Reported on page no. |
| --- | --- | --- | --- | --- |
|  | **Title and abstract** | | |  |
|  | Title and structured abstract | 1a | Identification as a randomised trial | 1, 3 |
|  |  | 1b | Structured summary of the trial design, methods, results, and conclusions | 3 |
|  | **Open science** | | |  |
|  | Trial registration | 2 | Name of trial registry, identifying number (with URL) and date of registration | 3, 16 |
|  | Protocol and statistical analysis plan | 3 | Where the trial protocol and statistical analysis plan can be accessed | 8 |
|  | Data sharing | 4 | Where and how the individual de-identified participant data (including data dictionary), statistical code and any other materials can be accessed | 8, 16 |
|  | Funding and conflicts of interest | 5a | Sources of funding and other support (eg, supply of drugs), and role of funders in the design, conduct, analysis and reporting of the trial | 16 |
|  |  | 5b | Financial and other conflicts of interest of the manuscript authors | 16 |
|  | **Introduction** | | |  |
|  | Background and rationale | 6 | Scientific background and rationale | 4 |
|  | Objectives | 7 | Specific objectives related to benefits and harms | n.a. |
|  | **Methods** | | |  |
|  | Patient and public involvement | 8 | Details of patient or public involvement in the design, conduct and reporting of the trial | 5 |
|  | Trial design | 9 | Description of trial design including type of trial (eg, parallel group, crossover), allocation ratio, and framework (eg, superiority, equivalence, non-inferiority, exploratory) | 5 |
|  | Changes to trial protocol | 10 | Important changes to the trial after it commenced including any outcomes or analyses that were not prespecified, with reason | 7 |
|  | Trial setting | 11 | Settings (eg, community, hospital) and locations (eg, countries, sites) where the trial was conducted | 5 |
|  | Eligibility criteria | 12a | Eligibility criteria for participants | 5 |
|  |  | 12b | If applicable, eligibility criteria for sites and for individuals delivering the interventions (eg, surgeons, physiotherapists) | 5 |
|  | Intervention and comparator | 13 | Intervention and comparator with sufficient details to allow replication. If relevant, where additional materials describing the intervention and comparator (eg, intervention manual) can be accessed | 5 |
|  | Outcomes | 14 | Prespecified primary and secondary outcomes, including the specific measurement variable (eg, systolic blood pressure), analysis metric (eg, change from baseline, final value, time to event), method of aggregation (eg, median, proportion), and time point for each outcome | 5 to 7 |
|  | Harms | 15 | How harms were defined and assessed (eg, systematically, non-systematically) | n.a. |
|  | Sample size | 16a | How sample size was determined, including all assumptions supporting the sample size calculation | 7 |
|  |  | 16b | Explanation of any interim analyses and stopping guidelines | 7 |
|  | Randomisation: |  |  |  |
|  | Sequence generation | 17a | Who generated the random allocation sequence and the method used | 7 |
|  |  | 17b | Type of randomisation and details of any restriction (eg, stratification, blocking and block size) | 5 |
|  |  |  |  | **Reported on page no.** |
|  | Allocation concealment mechanism | 18 | Mechanism used to implement the random allocation sequence (eg, central computer/telephone; sequentially numbered, opaque, sealed containers), describing any steps to conceal the sequence until interventions were assigned | 5 |
|  | Implementation | 19 | Whether the personnel who enrolled and those who assigned participants to the interventions had access to the random allocation sequence | 5, 16 |
|  | Blinding | 20a | Who was blinded after assignment to interventions (eg, participants, care providers, outcome assessors, data analysts) | 5 |
|  |  | 20b | If blinded, how blinding was achieved and description of the similarity of interventions | 5 |
|  | Statistical methods | 21a | Statistical methods used to compare groups for primary and secondary outcomes, including harms | 7 |
|  |  | 21b | Definition of who is included in each analysis (eg, all randomised participants), and in which group | 7 |
|  |  | 21c | How missing data were handled in the analysis | 7 |
|  |  | 21d | Methods for any additional analyses (eg, subgroup and sensitivity analyses), distinguishing prespecified from post hoc | 7 |
|  | **Results** | | |  |
|  | Participant flow, including flow diagram | 22a | For each group, the numbers of participants who were randomly assigned, received intended intervention, and were analysed for the primary outcome | 9 |
|  |  | 22b | For each group, losses and exclusions after randomisation, together with reasons | 9, 18 |
|  | Recruitment | 23a | Dates defining the periods of recruitment and follow-up for outcomes of benefits and harms | 3, 9 |
|  |  | 23b | If relevant, why the trial ended or was stopped | n.a. |
|  | Intervention and comparator delivery | 24a | Intervention and comparator as they were actually administered (eg, where appropriate, who delivered the intervention/comparator, how participants adhered, whether they were delivered as intended (fidelity)) | 9 |
|  |  | 24b | Concomitant care received during the trial for each group | 9 |
|  | Baseline data | 25 | A table showing baseline demographic and clinical characteristics for each group | 22 |
|  | Numbers analysed,  outcomes and estimation | 26 | For each primary and secondary outcome, by group:  ● the number of participants included in the analysis  ● the number of participants with available data at the outcome time point  ● result for each group, and the estimated effect size and its precision (such as 95% confidence interval)  ● for binary outcomes, presentation of both absolute and relative effect size | 20, 23 to 28 |
|  | Harms | 27 | All harms or unintended events in each group | n.a. |
|  | Ancillary analyses | 28 | Any other analyses performed, including subgroup and sensitivity analyses, distinguishing pre-specified from post hoc | 9, 10 |
|  | **Discussion** | | |  |
|  | Interpretation | 29 | Interpretation consistent with results, balancing benefits and harms, and considering other relevant evidence | 11, 12 |
|  | Limitations | 30 | Trial limitations, addressing sources of potential bias, imprecision, generalisability, and, if relevant, multiplicity of analyses | 11, 12 |

APPENDIX 4: CHEERS statement checklist

| **Topic** | **No.** | **Item** | **Location where item is reported** |
| --- | --- | --- | --- |
| **Title** |  |  |  |
|  | 1 | Identify the study as an economic evaluation and specify the interventions being compared. | 1 |
| **Abstract** |  |  |  |
|  | 2 | Provide a structured summary that highlights context, key methods, results, and alternative analyses. | 3 |
| **Introduction** |  |  |  |
| **Background and objectives** | 3 | Give the context for the study, the study question, and its practical relevance for decision making in policy or practice. | 4 |
| **Methods** |  |  |  |
| **Health economic analysis plan** | 4 | Indicate whether a health economic analysis plan was developed and where available. | 5 |
| **Study population** | 5 | Describe characteristics of the study population (such as age range, demographics, socioeconomic, or clinical characteristics). | 5 |
| **Setting and location** | 6 | Provide relevant contextual information that may influence findings. | 5 |
| **Comparators** | 7 | Describe the interventions or strategies being compared and why chosen. | 5 |
| **Perspective** | 8 | State the perspective(s) adopted by the study and why chosen. | 6 |
| **Time horizon** | 9 | State the time horizon for the study and why appropriate. | 6 |
| **Discount rate** | 10 | Report the discount rate(s) and reason chosen. | n.a. |
| **Selection of outcomes** | 11 | Describe what outcomes were used as the measure(s) of benefit(s) and harm(s). | 5,6 |
| **Measurement of outcomes** | 12 | Describe how outcomes used to capture benefit(s) and harm(s) were measured. | 5,6 |
| **Valuation of outcomes** | 13 | Describe the population and methods used to measure and value outcomes. | 6 |
| **Measurement and valuation of resources and costs** | 14 | Describe how costs were valued. | 6 |
| **Currency, price date, and conversion** | 15 | Report the dates of the estimated resource quantities and unit costs, plus the currency and year of conversion. | 6 |
| **Rationale and description of model** | 16 | If modelling is used, describe in detail and why used. Report if the model is publicly available and where it can be accessed. | 6,7 |
| **Analytics and assumptions** | 17 | Describe any methods for analysing or statistically transforming data, any extrapolation methods, and approaches for validating any model used. | 6,7 |
| **Characterising heterogeneity** | 18 | Describe any methods used for estimating how the results of the study vary for subgroups. | 6,7 |
| **Characterising distributional effects** | 19 | Describe how impacts are distributed across different individuals or adjustments made to reflect priority populations. | 7 |
| **Characterising uncertainty** | 20 | Describe methods to characterise any sources of uncertainty in the analysis. | 6,7 |
| **Approach to engagement with patients and others affected by the study** | 21 | Describe any approaches to engage patients or service recipients, the general public, communities, or stakeholders (such as clinicians or payers) in the design of the study. | 6,7 |
| **Results** |  |  |  |
| **Study parameters** | 22 | Report all analytic inputs (such as values, ranges, references) including uncertainty or distributional assumptions. | 9 |
| **Summary of main results** | 23 | Report the mean values for the main categories of costs and outcomes of interest and summarise them in the most appropriate overall measure. | 9,10 |
| **Effect of uncertainty** | 24 | Describe how uncertainty about analytic judgments, inputs, or projections affect findings. Report the effect of choice of discount rate and time horizon, if applicable. | 9 |
| **Effect of engagement with patients and others affected by the study** | 25 | Report on any difference patient/service recipient, general public, community, or stakeholder involvement made to the approach or findings of the study | n.a. |
| **Discussion** |  |  |  |
| **Study findings, limitations, generalisability, and current knowledge** | 26 | Report key findings, limitations, ethical or equity considerations not captured, and how these could affect patients, policy, or practice. | 11 |
| **Other relevant information** |  |  |  |
| **Source of funding** | 27 | Describe how the study was funded and any role of the funder in the identification, design, conduct, and reporting of the analysis | 16 |
| **Conflicts of interest** | 28 | Report authors conflicts of interest according to journal or International Committee of Medical Journal Editors requirements. | 16 |

*From:* Husereau D, Drummond M, Augustovski F, et al. Consolidated Health Economic Evaluation Reporting Standards 2022 (CHEERS 2022) Explanation and Elaboration: A Report of the ISPOR CHEERS II Good Practices Task Force. Value Health 2022;25. <doi:10.1016/j.jval.2021.10.008>

APPENDIX 5: Resource utilisation per country

| **TABLE I \|** Intra- and Postoperative Resource Utilisation, per-country | | | | | | | | | | | | | | | | | |
| --- | --- | --- | --- | --- | --- | --- | --- | --- | --- | --- | --- | --- | --- | --- | --- | --- | --- |
| **Resource use**  **item†** | **The Netherlands** | | **Germany** | | **Belgium** | | | | **England** | | | | | **Italy** | | **Norway** | |
|  | **OH**  **(n=20)** | **LH**  **(n=20)** | **OH**  **(n=3)** | **LH**  **(n=4)** | **OH**  **(n=36)** | | **LH**  **(n=36)** | | **OH**  **(n=59)** | | | | **LH**  **(n=56)** | **OH**  **(n=41)** | **LH**  **(n=44)** | **OH**  **(n=7)** | **LH**  **(n=6)** |
| **INTRA-OPERATIVE** | Mean (SD) | | | | |  | |  | |  |  | | | | | | |
| Cutting time, minutes | 252  (52)  n=20 | 360 (117)  n=20 | 292  (110)  n=3 | 322  (55)  n=4 | 236  (92)  n=36 | | 270  (86)  n=36 | | 226  (73)  n=56 | | | | 343  (91)  n=52 | 299  (84)  n=40 | 314  (80)  n=44 | 223  (55)  n=5 | 227  (69)  n=6 |
| Sitting time, minutes | 355  (71)  n=12 | 450  (119)  n=11 | 401  (102)  n=3 | 440  (64)  n=3 | 363  (90)  n=34 | | 391  (86)  n=33 | | 322  (67)  n=43 | | | | 462  (93)  n=35 | 392  (86)  n=40 | 400  (86)  n=44 | 332  (42)  n=7 | 321  (42)  n=4 |
| Transfusion requirement, units | 0  (0)  n=20 | 0·2  (0·894)  n=20 | 11  (15·6)  n=2 | 1  (1·155)  n=4 | 0·31  (0·99)  n=36 | | 0·139  (0·494)  n=36 | | 0·02  (0·131)  n=59 | | | | 0·255  (0·788)  n=56 | 0·15  (0·483)  n=41 | 0·273  (0·694)  n=44 | 0·57  (0·976)  n=7 | 0  (0)  n=6 |
| **POST-OPERATIVE** | Median (IQR) | | | | | | | | | | | | | | | | |
| Length of stay, days | 6  (5-7)  n=20 | 7  (3-9)  n=20 | 7  (0-7)  n=3 | 8  (7-11)  n=4 | 6  (5-7)  n=36 | | 4  (3-6)  n=36 | | 6  (5-8)  n=59 | | | | 5  (4-6)  n=56 | 6  (5-7)  n=41 | 5  (4-8)  n=44 | 6  (4-7)  n=7 | 3  (2-3)  n=6 |
| Time to oral analgesia, days | 3  (3-4)  n=19 | 2  (2-3)  n=20 | 4  (1-4)  n=2 | 2  (0-3)  n=4 | 3  (2-4)  n=33 | | 2  (1-3)  n=35 | | 4  (3-5)  n=58 | | | | 3  (2-3)  n=55 | 3  (2-3)  n=40 | 2  (2-3)  n=44 | 5  (2-7)  n=7 | 2  (1-3)  n=6 |
| Laboratory blood sampling, days | 3  (2-4)  n=20 | 3  (2-4)  n=20 | 4  (0-4)  n=3 | 4  (4-4)  n=4 | 4  (3-4)  n=36 | | 3  (2-4)  n=36 | | 4  (4-4)  n=59 | | | | 4  (3-4)  n=56 | 3  (2-4)  n=41 | 3  (3-4)  n=44 | 4  (4-5)  n=7 | 3  (2-3)  n=6 |
| 90-Day Readmission, n (%) | 4 (21)  n=19 | 9 (50)  n=18 | 1 (33)  n=3 | 0 (0)  n=4 | 3 (9)  n=34 | | 6 (18)  n=33 | | 11 (21)  n=52 | | | | 9 (17)  n=53 | 0 (0)  n=38 | 2 (5)  n=42 | 1 (17)  n=6 | 3 (50)  n=6 |
| Readmission length, days | 8(6-17)  n=4 | 26 (7-26)  n=2 | 9 (9-9)  n=1 | - | 10 (6-10)  n=3 | | 5(3-9)  n=6 | | 6 (1-10)  n=11 | | | | 5(1-9)  n=10 | - | 4(3-4)  n=2 | 15(15-15)  n=1 | 7(2-7)  n=3 |
| **3-MONTHS*** | Mean (SD) / Total | | | | | | | | | | |  | | | | | |
| ER consults | 0·2 (0·4) / 2  n=9 | 0·0 (0·0) / 0  n=8 | 1·5 (2·1) / 3  n=2 | 0·0 (0·0) / 0  n=4 | 0·0 (0·2) / 4  n=29 | | 0·0 (0·2) / 1  n=30 | | 0·2 (0·5) / 8  n=40 | | | | 0·2 (0·5) / 8  n=36 | 0·0 (0·0) / 0  n=25 | 0·1 (0·4) / 2  n=29 | 0·3 (0·8) / 2  n=6 | 0·8 (0·8) / 4  n=5 |
| GP consults | 1·4 (1·9) / 13  n=9 | 0·3 (0·8) / 2  n=7 | 3 (0)  / 6  n=2 | 2·5 (3·8) / 10  n=4 | 2·0 (2·3) / 57  n=29 | | 1·0 (0·3) / 29  n=30 | | 1·7 (2·7) / 66  n=39 | | | | 0·8 (1·1) / 28  n=44 | 0·7 (1·1) / 16  n=24 | 0·8 (1·2) / 20  n=26 | 0·8 (1·0) / 5  n=6 | 2·0 (2·1) / 10  n=5 |
| Specialist consults | 3·3 (3·5) / 30  n=9 | 1·3 (1·6) / 10  n=8 | 3·5 (5·0) / 7  n=2 | 3·0 (3·8) / 12  n=4 | 1·4 (1·4) / 41  n=29 | | 2·0 (2·2) / 61  n=30 | | 1·4 (1·9) / 54  n=40 | | | | 1·5 (1·7) / 53  n=36 | 0·9 (0·9) / 22  n=25 | 1·1 (0·8) / 31  n=28 | 1·2 (1·8) / 7  n=6 | 1·4 (1·1) / 7  n=5 |
| **6-MONTHS*** | Mean (SD) / Total | | | | | | | | | | | | | | | | |
| ER consults | 0·1 (0·3) / 1  n=9 | 0·3 (0·5) / 2  n=6 | 3·0 (0·0) / 3  n=1 | 0·0 (0·0) / 0  n= 2 | 0·0 (0·2) / 1  n=29 | | 0·0 (0·2) / 1  n=29 | | 0·1 (0·6) / 5  n= 39 | | | | 0·1 (0·4) / 3  n=39 | 0·0 (0·0) / 0  n= 16 | 0·0 (0·0) / 0  n= 18 | 0·4 (0·9) / 2  n=5 | 0·8 (1·0) / 3  n= 4 |
| GP consults | 2·0 (2·5) / 18  n= 11 | 0·5 (0·8) / 3  n=6 | 3·0 (0·0)  / 3  n=1 | 0·0 (0·0) / 0  n=2 | 0·8 (1·7) / 25  n=29 | | 1·3 (2·5) / 35  n=27 | | 0·7 (1·9) / 28  n=38 | | | | 0·5 (0·9) / 17  n=37 | 0·3 (0·9) / 5  n=15 | 0·5 (1·1) / 8  n=16 | 1·0 (1·0) / 5  n=5 | 0·8 (1·0) / 3  n=4 |
| Specialist consults | 2·2 (4·5) / 20  n= 9 | 1·7 (3·1) / 10  n=6 | 3·0 (0·0) / 3  n=1 | 0·0 (0·0) / 0  n=2 | 1·5 (1·9) / 43  n=29 | | 1·6 (1·8) / 45  n=29 | | 0·5 (1·3) / 18  n=39 | | | | 0·8 (1·1) / 30  n=39 | 0·9 (1·1) / 15  n=17 | 0·8 (0·8) / 16  n=19 | 4·0 (6·9) / 20  n=5 | 2·0 (2·8) / 8  n=4 |
| **12-MONTHS*** | Mean (SD) / Total | | | | | | | | | | | | | | | | |
| ER consults | 0·0 (0·0) / 0  n=6 | 0·5 (1·0) / 2  n=4 | 0·0 (0·0) / 0  n=1 | 0·0 (0·0) / 0  n=1 | 0·0 (0·0) / 0  n=26 | | 0·0 (0·0) / 0  n=27 | | 0·0 (0·2) / 1  n=30 | | | | 0·0 (0·2) / 1  n=35 | 0·0 (0·0) / 0  n=14 | 0·0 (0·0) / 0  n=20 | 0·0 (0·0) / 0  n=4 | 0·3 (0·5) / 0  n=4 |
| GP consults | 0·0 (0·0) / 0  n=6 | 0·5 (1·0) / 2  n=4 | 0·0 (0·0) / 0  n=1 | 0·0 (0·0) / 0  n=1 | 0·0 (0·0) / 0  n=26 | | 0·0 (0·0) / 0  n=27 | | 0·0 (0·2) / 1  n=30 | | | | 0·0 (0·2) / 1  n=35 | 0·0 (0·0) / 0  n=14 | 0·0 (0·0) / 0  n=20 | 0·0 (0·0) / 0  n=4 | 0·3 (0·5) / 0  n=4 |
| Specialist consults | 0·0 (0·0) / 0  n=6 | 0·5 (1·0) / 2  n=4 | 0·0 (0·0) / 0  n=1 | 0·0 (0·0) / 0  n=1 | 0·0 (0·0) / 0  n=26 | | 0·0 (0·0) / 0  n=27 | | 0·0 (0·2) / 1  n=30 | | | | 0·0 (0·2) / 1  n=35 | 0·0 (0·0) / 0  n=14 | 0·0 (0·0) / 0  n=20 | 0·0 (0·0) / 0  n=4 | 0·3 (0·5) / 0  n=4 |
| LH, Laparoscopic; OH, Open hemihepatectomy. *Patient reported outcome †Resource utilisation is presented as intention to treat, units are total quantities unless stated otherwise. | | | | | | | | | | | | | | | | | |

| ***(Table I continued)*** | | | | | | | | | | | | |
| --- | --- | --- | --- | --- | --- | --- | --- | --- | --- | --- | --- | --- |
| **Resource use**  **item†** | **The Netherlands** | | **Germany** | | **Belgium** | | **England** | | **Italy** | | **Norway** | |
|  | **OH**  **(n=20)** | **LH**  **(n=20)** | **OH**  **(n=3)** | **LH**  **(n=4)** | **OH**  **(n=36)** | **LH**  **(n=36)** | **OH**  **(n=59)** | **LH**  **(n=56)** | **OH**  **(n=41)** | **LH**  **(n=44)** | **OH**  **(n=7)** | **LH**  **(n=6)** |
| **ADVERSE-EVENT INTERVENTIONS** | | | | | | | | | | | | |
| Medicatory |  |  |  |  |  |  |  |  |  |  |  |  |
| Antibiotics | 3 | 4 | 1 | 0 | 10 | 7 | 27 | 9 | 1 | 7 | 0 | 2 |
| Thrombotic events | 1 | 3 | 0 | 0 | 0 | 0 | 2 | 0 | 0 | 0 | 0 | 0 |
| Transfusions | 5 | 2 | 1 | 0 | 0 | 0 | 7 | 5 | 1 | 1 | 0 | 0 |
| CPR | 0 | 0 | 0 | 0 | 1 | 0 | 0 | 0 | 0 | 0 | 0 | 0 |
| CD-II not specified | 0 | 0 | 0 | 0 | 0 | 0 | 0 | 2 | 1 | 1 | 0 | 0 |
| Radiologic |  |  |  |  |  |  |  |  |  |  |  |  |
| US-guided drainage | 0 | 2 | 0 | 0 | 6 | 2 | 9 | 5 | 1 | 0 | 1 | 3 |
| CT-guided drainage | 0 | 0 | 0 | 0 | 0 | 2 | 0 | 0 | 0 | 0 | 0 | 0 |
| Endoscopic stent | 0 | 0 | 0 | 0 | 0 | 0 | 0 | 0 | 1 | 0 | 0 | 0 |
| ERCP | 0 | 2 | 0 | 0 | 1 | 2 | 0 | 1 | 0 | 0 | 0 | 0 |
| Coiling of vessel | 0 | 1 | 0 | 0 | 0 | 0 | 0 | 0 | 0 | 0 | 0 | 0 |
| PTC drain | 0 | 1 | 0 | 0 | 0 | 0 | 0 | 0 | 0 | 0 | 0 | 0 |
| C-D III not specified | 2 | 1 | 0 | 0 | 0 | 0 | 0 | 2 | 0 | 1 | 0 | 0 |
| Operation |  |  |  |  |  |  |  |  |  |  |  |  |
| Local anaesthesia | 0 | 0 | 0 | 0 | 1 | 0 | 0 | 0 | 0 | 0 | 0 | 1 |
| General anaesthesia | 1 | 1 | 1 | 0 | 1 | 1 | 5 | 1 | 0 | 1 | 0 | 0 |
| Intensive Care |  |  |  |  |  |  |  |  |  |  |  |  |
| Admission | 3 | 2 | 1 | 0 | 7 | 2 | 29 | 24 | 1 | 4 | 0 | 0 |
| C-D, Clavien-Dindo grade; CT, computed-tomography; ERCP, endoscopic retrograde cholangiopancreatography;IQR, interquartile range; LH, Laparoscopic; OH, Open hemihepatectomy; US, ultrasound; PTC, percutaneous transhepatic cholangiogram, SD; standard deviation. *Patient reported outcome †Resource utilization is presented as intention to treat, units are total quantities unless stated otherwise. | | | | | | | | | | | | |

| **TABLE II \|** Resource Utilisation of Operative Disposables, per-country | | | | | | | |  | | | | | |
| --- | --- | --- | --- | --- | --- | --- | --- | --- | --- | --- | --- | --- | --- |
| **Resource use**  **Item*** | **The Netherlands** | | **Germany** | | **Belgium** | | **England** | | | **Italy** | | **Norway** | |
|  | **OH**  **(n=20)** | **LH**  **(n=20)** | **OH**  **(n=3)** | **LH**  **(n=4)** | **OH**  **(n=36)** | **LH**  **(n=36)** | **OH**  **(n=59)** | | **LH**  **(n=56)** | **OH**  **(n=41)** | **LH**  **(n=44)** | **OH**  **(n=7)** | **LH**  **(n=6)** |
| **ANAESTHESIA** |  | | | | | | | | | | | | |
|  | **n=20** | **n=20** | **n=2** | **n=4** | **n=36** | **n=35** | **n=58** | | **n=53** | **n=40** | **n=44** | **n=7** | **n=5** |
| Epidural | 8 | 5 | 0 | 0 | 7 | 7 | 30 | | 21 | 22 | 20 | 0 | 0 |
| Spinal/  Paravertebral | 0 | 0 | 0 | 0 | 2 | 2 | 0 | | 3 | 13 | 14 | 0 | 0 |
|  | **n=20** | **n=20** | **n=3** | **n=4** | **n=36** | **n=35** | **n=59** | | **n=53** | **n=40** | **n=44** | **n=7** | **n=5** |
| Arterial line | 20 | 19 | 2 | 3 | 35 | 35 | 59 | | 53 | 39 | 43 | 7 | 5 |
| Central venous line | 14 | 16 | 2 | 3 | 36 | 34 | 58 | | 51 | 5 | 11 | 7 | 5 |
| **SURGICAL** | | | | | | | | | | | | | |
|  | **n=17** | **n=17** | **n=0** | **n=0** | **n=36** | **n=35** | **n=48** | | **n=35** | **n=0** | **n=0** | **n=7** | **n=5** |
| CUSA | 15 | - |  |  | 35 | 5 | 45 | | 3 |  |  | 3 | - |
| Laparoscopic CUSA | - | 18 |  |  | - | 28 | - | | 28 |  |  | - | 1 |
| Argon | 2 | 0 |  |  | 9 | 24 | 45 | | 5 |  |  | 0 | 0 |
| Staplers and reloads | 36 | 65 |  |  | 93 | 247 | 110 | | 127 |  |  | 40 | 38 |
| Electrothermal tissue sealers | 0 | 21 |  |  | 2 | 54 | 1 | | 26 |  |  | 6 | 9 |
| Trocars | - | 107 |  |  | - | 180 | - | | 152 |  |  | - | 20 |
|  | **n=20** | **n=19** | **n=3** | **n=3** | **n=36** | **n=35** | **n=50** | | **n=41** | **n=40** | **n=43** | **n=7** | **n=5** |
| Haemostatic Sealant | 20 | 19 | 0 | 0 | 57 | 31 | 53 | | 53 | 9 | 15 | 6 | 1 |
| CUSA, cavitronic ultrasonic surgical aspirator; LH, Laparoscopic; OH, Open hemihepatectomy. *Resource utilisation is displayed as intention to treat and units are total quantities unless stated otherwise. | | | | | | | | | | | | | |

| **TABLE III** \| Mean Observed EQ-5D-3L Utility per Country | | | | | | | | | | | | |
| --- | --- | --- | --- | --- | --- | --- | --- | --- | --- | --- | --- | --- |
| **Follow-up timepoint** | **The Netherlands** | | **Germany** | | **Belgium** | | **England** | | **Italy** | | **Norway** | |
|  | **OH**  **(n=20)** | **LH**  **(n=20)** | **OH**  **(n=3)** | **LH**  **(n=4)** | **OH**  **(n=36)** | **LH**  **(n=36)** | **OH**  **(n=59)** | **LH**  **(n=56)** | **OH**  **(n=41)** | **LH**  **(n=44)** | **OH**  **(n=7)** | **LH**  **(n=6)** |
|  |  |  |  | | | | | | | | | |
| Baseline | 0·815  n=20 | 0·783  n=20 | 0·859  n=3 | 0·875  n=4 | 0·750  n=36 | 0·766  n=36 | 0·817  n=59 | 0·845  n=54 | 0·982  n=40 | 0·958  n=44 | 0·962  n=7 | 0·871  n=6 |
|  |  |  |  |  |  |  |  |  |  |  |  |  |
| Discharge | 0·589  n=15 | 0·554  n=15 | 0·850  n=2 | 0·893  n=4 | 0·554  n=31 | 0·688  n=30 | 0·440  n=43 | 0·606  n=48 | 0·741  n=38 | 0·780  n=42 | 0·795  n=4 | 0·720  n=3 |
|  |  |  |  |  |  |  |  |  |  |  |  |  |
| 10-day | 0·775  n=14 | 0·748  n=13 | 0·788  n=2 | 0·796  n=3 | 0·477  n=32 | 0·663  n=33 | 0·603  n=41 | 0·707  n=41 | 0·853  n=37 | 0·870  n=43 | 0·795  n=4 | 0·720  n=3 |
|  |  |  |  |  |  |  |  |  |  |  |  |  |
| 3-month | 0·741  n=10 | 0·746  n=10 | 0·844  n=2 | 0·943  n=4 | 0·738  n=30 | 0·822  n=30 | 0·731  n=42 | 0·797  n=40 | 0·953  n=38 | 0·956  n=42 | 0·932  n=6 | 0·791  n=5 |
|  |  |  |  |  |  |  |  |  |  |  |  |  |
| 6-month | 0·695  n=10 | 0·637  n=8 | 0·788  n=1 | 1·000  n=2 | 0·679  n=31 | 0·778  n=27 | 0·734  n=40 | 0·784  n=42 | 0·964  n=37 | 0·945  n=41 | 0·974  n=5 | 0·834  n=4 |
|  |  |  |  |  |  |  |  |  |  |  |  |  |
| 12-month | 0·619  n=7 | 0·591  n=6 | 0·394  n=2 | 1·000  n=1 | 0·639  n=30 | 0·711  n=29 | 0·683  n=33 | 0·732  n=40 | 0·937  n=36 | 0·936  n=39 | 0·800  n=5 | 0·843  n=4 |
| LH, Laparoscopic; OH, Open hemihepatectomy. After death utility score was assumed to be 0. | | | | | | | | | | | | |

APPENDIX 6: Sensitivity Analysis - Dutch EQ-5D-3L value set

| **TABLE I**\| Imputed mean EQ-5D-3L utility scores, using the Dutch index for all patients | | |
| --- | --- | --- |
| Follow-up time point | OH  n=166 | LH  n=166 |
| Baseline | 0·859 (0·217) | 0·860 (0·198) |
| Discharge | 0·620 (0·267) | 0·698 (0·239) |
| 10-day follow-up | 0·689 (0·276) | 0·769 (0·221) |
| 3-month follow-up | 0·785 (0·275) | 0·831 (0·241) |
| 6-month follow-up | 0·765 (0·306) | 0·789 (0·269) |
| 12-month follow-up | 0·723 (0·332) | 0·770 (0·299) |
|  |  |  |
| QALYs | 0·747 (0·261) | 0·788 (0·229) |
| LH, laparoscopic hemihepatectomy; OH, open hemihepatectomy. Values are mean (SD), unless indicated otherwise. | | |

| FIGURE I \| Cost-effectiveness sensitivity analysis |
| --- |
| 1. Cost-effectiveness plane   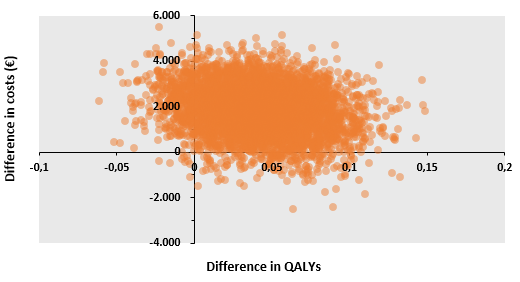 |
| b. Cost-effectiveness acceptability curve  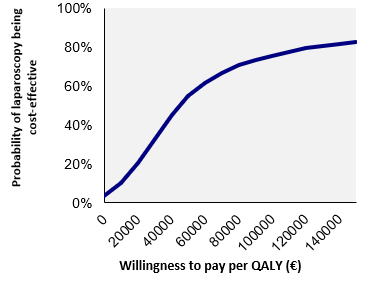 |
| a. Cost effectiveness plane for 5000 bootstrapped repetitions showing differences in total costs and QALYs between open and laparoscopic hemihepatectomy, when Dutch EQ-5D-3L value sets are applied to all patients. A majority of the datapoints lie in the right upper quadrant demonstrating laparoscopic hemihepatectomy is more effective but more costly.  b. Cost-effectiveness acceptability curve showing the probability of laparoscopic hemihepatectomy being more cost-effective for various willingness to pay thresholds per QALY, when Dutch EQ-5D-3L value sets are applied to all patients. |

APPENDIX 7: ORANGE II PLUS collaborative

| **ORANGE II PLUS collaborative** | | |
| --- | --- | --- |
| **Centre** | **Name** | **Role** |
| Aachen University Hospital  Aachen, Germany | Ulf Neumann | Principal investigator |
|  | Florian Ulmer | Medical staff involved in patient care |
|  | Finja Clausen | Research nurse |
| Aintree University Hospital NHS Foundation Trust  Aintree, United Kingdom | Rafael Díaz-Nieto | Principal investigator |
|  | Michelle Lintforth | Research nurse |
| Amsterdam University Medical Centres  Amsterdam, The Netherlands | Marc Besselink | Principal investigator |
|  | Pieter Tanis | Medical staff involved in patient care |
|  | Burak Gorçek | PhD candidate |
|  | Marcel van der Poel | PhD candidate |
| University Hospitals Birmingham NHS Trust  Birmingham, United Kingdom | Robert Sutcliffe | Principal investigator |
|  | Ravi Marudanayagam | Medical staff involved in patient care |
|  | Penelope Rogers | Research nurse |
| Erasmus Hospital, Brussels, Belgium | Valerio Lucidi | Principal investigator |
|  | Viviane van Laethem | Research nurse |
| Ghent University Hospital  Ghent, Belgium | Roberto Troisi | Principal investigator |
|  | Frederik Berrevoet | Medical staff involved in patient care |
|  | Vincenzo Scuderi | Medical staff involved in patient care |
|  | Aude Vanlander | Medical staff involved in patient care |
|  | Betsy van Loo | Research nurse, trial coordinator |
|  | Kathleen Segers | Research nurse |
| Jessa Hospital  Hasselt, Belgium | Gregory Sergeant | Principal investigator |
| Groeninge General Hospital Kortrijk, Belgium | Mathieu D’Hondt | Principal investigator |
|  | Celine Demeyere | Research nurse |
| King’s College Hospital NHS Foundation Trust  London, United Kingdom | Krishna Menon | Principal investigator |
|  | Ane Zamalloa | Research nurse |
| Maastricht University Medical Centre+  Maastricht, The Netherlands | Ronald van Dam | Principal investigator, trial coordinator |
|  | Cornelis Dejong | Medical staff involved in patient care |
|  | Lloyd Brandts | Trial statistician |
|  | Robert Fichtinger | PhD candidate, trial coordinator |
|  | Bram Olij | PhD candidate |
|  | Merel Kimman | Health economics and Quality of life expert |
|  | Remon Korenblik | PhD candidate |
|  | Gabriela Pilz da Cunha | PhD candidate |
| Maastricht University  Maastricht, The Netherlands | Gerard van Breukelen | Trial statistician |
| San Raffaele Hospital Milan, Italy | Luca Aldrighetti | Principal investigator |
|  | Francesca Ratti | Medical staff involved in patient care |
| Newcastle upon Tyne Hospitals NHS Foundation Trust  Newcastle, United Kingdom | Steve White | Principal investigator |
|  | Stuart Robinson | Medical staff involved in patient care |
|  | Caroline Brunton | Research nurse |
| Oslo University Hospital  Oslo, Norway | Björn Edwin | Principal investigator |
|  | Åsmund Fretland | Medical staff involved in patient care |
|  | Davit Aghayan | PhD candidate |
| Oxford University Hospitals NHS Foundation Trust  Oxford, United Kingdom | Zahir Soonawalla | Principal investigator |
|  | Katherine Gordon-Quayle | Research nurse |
| University Hospitals Plymouth NHS Foundation Trust  Plymouth, United Kingdom | Somaiah Aroori | Principal investigator |
|  | Tracy Ward | Research nurse |
| University Hospital Southampton NHS Foundation Trust  Southampton, United Kingdom | John Primrose | Principal investigator |
|  | Mohammad Abu Hilal | Principal investigator |
|  | Christopher Kümmerli | PhD candidate |
|  | Jess Boxal | Research nurse |
| Southampton Clinical Trials Unit  Southampton, United Kingdom | Zina Eminton | Trial coordinator |
